# Metastatic Malignant Melanoma of the Inguinal Lymph Node with Unknown Primary Lesion

**DOI:** 10.1155/2015/879460

**Published:** 2015-02-01

**Authors:** Sherif Ali Eltawansy, Ryane Panasiti, Samaa Hasanien, Dennis Lourdusamy, David Sharon

**Affiliations:** ^1^Department of Internal Medicine, Monmouth Medical Center, Long Branch, NJ 07740, USA; ^2^Department of Pathology, Monmouth Medical Center, Long Branch, NJ 07740, USA; ^3^Department of Medicine, Cairo University Medical School, Cairo 11562, Egypt; ^4^Department of Oncology, Monmouth Medical Center, Long Branch, NJ 07740, USA

## Abstract

*Background*. Malignant melanoma could present with metastasis with unknown primary (MUP) and this happens in 2-3% according to the studies. Around 90% of melanomas have cutaneous origin, but still there are melanomas that could be found in visceral organs or lymph nodes with unknown primary site. Spontaneous regression of the primary site could be an explanation. *Case Report*. We report a 58-year-old Caucasian male who presented with a right sided swelling in the inguinal region. Surgery was performed and biopsy showed metastatic malignant melanoma. No cutaneous lesions were identified by history or physical examination. Work up could not detect the primary lesion and patient was started on radiotherapy and immunotherapy. *Conclusion*. We present a case of malignant melanoma of unknown primary presenting in an unusual place which is the inguinal lymph node. Theories try to explain the pathway of development of such tumors and one of the theories mentions that it could be a spontaneous regression of the primary cutaneous lesion. Another theory is that it could be from transformation of aberrant melanocyte within the lymph node. Prognosis is postulated to be better in this case than in melanoma with a known primary.

## 1. Case Presentation

**Figure 1:**
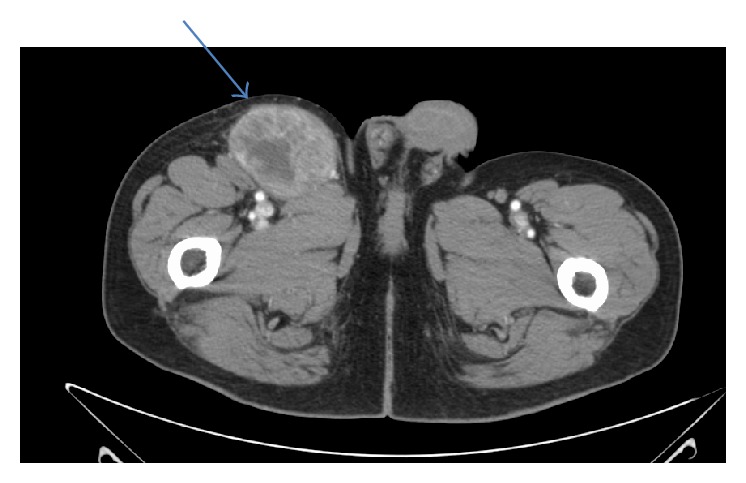
*Technique*. Axial images were obtained from the lower chest to the pubic symphysis with intravenous and oral contrast. Patient received 125 milliliters of Omnipaque 350 as a contrast agent.* Findings*. Lymph nodes: a necrotic appearing, enlarged right inguinal lymph node is noted, measuring 5.0 × 7.1 cm (blue arrow). Multiple other right inguinal lymph nodes are noted, borderline in size. An enlarged right external iliac lymph node is seen on measuring 2.0 × 1.5 cm. A mildly enlarged 1.5 × 1.1 cm right external iliac lymph node is seen just posterior to this. Nothing abnormal is detected in abdomen or pelvis worthy to mention.

**Figure 2:**
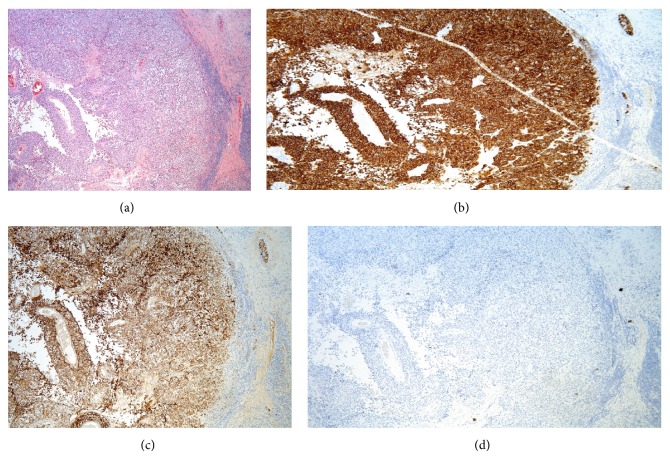
(a) Metastatic melanoma replacing a lymph node with a thin rim of residual lymphocytes (far right) H&E 5x. (b) The malignant cells are strongly positive for Melan A, (c) positive for HMB45, and (d) negative for cytokeratin AE1/AE3 (IHC, 5x). Although this staining pattern could be seen in benign and malignant melanocytes, its presence in a lymph node indicates metastasis.

We report a case of a 58-year-old Caucasian male with no significant past medical history and also had no regular follow-up, came with slowly growing right sided inguinal mass. He did not remember any explanation or precipitating event to the swelling and he did not remember when swelling started to exist exactly but approximately over weeks. His surgical history included tonsillectomy. He was smoking for 40 years. He used to work in an office but recently retired and was living with family. On evaluation, there was no history of constitutional symptoms like fever, chills, and loss of energy or weight. Review of systems was completely negative. He came to the hospital. After physical examination, generalized lymphadenopathy was excluded after careful examination apart from that local right inguinal mass. The swelling was firm and nonfluctuant. It had an intact overlying skin with no redness or discharging tract. Blood work was unremarkable and HIV was nonreactive. CT scan of the abdomen and pelvis with oral and IV contrast was done (Figure 1) and showed necrotic appearing, enlarged right inguinal lymph node, measuring 5.0 × 7.1 cm. Additionally mildly enlarged right inguinal and external iliac lymph nodes were seen. Chest X-ray was unremarkable. Surgery was done with complete excision of the inguinal lymph node under general anesthesia. The iliac lymph nodes were not excised, being small and multiple. Pathology came back with malignant melanoma (Figures 2(a), 2(b), 2(c), and 2(d)). So the plan was to manage the remaining lymph nodes that were not excised with radiotherapy and (ipilimumab). He was discharged for follow-up in the clinic. Careful history and thorough physical examination were obtained, including examination of the lymph nodes and skin all over the body. Genital examination and digital rectal examination did not show any evidence for a primary lesion also. Patient noticed a fluctuant area in 3 the site of previous lymphadenopathy 1 week after the surgery. Using a 20-gauge needle, approximately 8 mL of seroma was evacuated. The fluid was serous in nature. There was a decrease in size in the fluctuant area. The fluid was drained with fine needle and aspiration fluid was sent for cytology coming back with malignant melanoma with the same pathology. PET scan was done and did not show any abnormal uptake all over the body. Patient was referred to a dermatologist for further evaluation. As a desperate trial, 2 lesions were excised. One was from his upper back of the trunk and the other was in the left thigh. Both looked like brown nonraised moles. Punch biopsy was obtained. The one from the back came back as junctional nevus and the other one from the left thigh came back as lentigo simplex. The case was then diagnosed as a metastatic melanoma of unknown primary. Patient received radiation therapy and then started on ipilimumab cycles.

## 2. Discussion

### 2.1. Metastatic Melanoma of Unknown Primary (MUP)

Our case represents an example of malignant melanoma with unknown primary (MUP). About 2-3% of all melanoma patients present with a melanoma metastasis without a detectable primary tumor [1]. Although the true etiology of an MUP is unknown, several explanations have been suggested and include (1) a concurrent, unrecognized melanoma; (2) a previously excised melanoma that was misdiagnosed either clinically or pathologically; (3) an antecedent, unrecognized, spontaneously regressed primary melanoma; and (4) the de novo malignant transformation of an aberrant melanocyte within a lymph node [2]. Benign nevus cells are commonly found in lymph nodes and other tissues, and melanomas arising from nevus cells in lymph nodes have been described [3, 4]. The previously noted clinical and histologic observations made during the previous three decades support the disappearance of a primary lesion as a result of spontaneous regression, which still remains a plausible explanation for MUP [5].

A retrospective analysis of patients with melanoma of unknown primary (MUP) was done on 2,485 melanoma patients; 65 (2.6%) had MUP. Thirty patients had lymph node metastases, 12 cutaneous or subcutaneous metastases, and 23 visceral metastases [1].

MUP patients comprise a heterogeneous group. Like patients with metastatic melanoma of a known primary (MKP), MUP patients can present with (sub)cutaneous, nodal metastasis, and/or metastasis to the viscera, bones, or brain. In patients with only (sub)cutaneous or nodal metastases it is impossible to differentiate between regional versus distant metastases, as the primary site is unknown. This distinction would be important for the assessment of prognosis [6]. Axillary nodal metastasis in patients with MUP is more common, but inguinal and cervical nodal metastases are the least common [2].

Work up for MUP should include full skin evaluation, brain imaging (CT or MRI), and CT imaging of the chest/abdomen and pelvis to rule out distant metastatic disease. Additional recommendations include otorhinolaryngological examinations to look for metastases to the head and neck region and proctoscopy and gynecologic examinations for patients with inguinal lymph node metastases. Ophthalmologic examinations should be reserved for patients who have MUP with visceral metastases, primarily of the liver [7]. In our reported case, full skin examination was done thoroughly and 2 skin lesions were sent for pathology and came back with lentigo simplex and junctional nevus. CT abdomen and pelvis beside chest X-ray did not show any evidence of primary or metastasis.

### 2.2. Prognosis of MUP

The relatively favorable long-term survival of patients with MUP supports the belief that, in the context of regional lymph node disease, MUP constitutes a manifestation of stage III disease rather than stage IV (M1a) distant lymph node disease. Therefore, patients who have metastatic melanoma in a regional node in the absence of a known primary site should undergo completion lymph node dissection. These patients also should be considered for adjuvant treatment trials that were designed for patients with stage III disease [8]. It is argued by several researchers that MUP patients have a better prognosis than MKP patients due to the same immunological mechanisms responsible for the regression of the primary tumour [9, 10]. The literature on the survival of MUP patients versus MKP patients, however, is not consistent. Moreover, Guiliano et al. were unable to identify differences in cellular immune responses between patients with a known or unknown primary melanoma [11]. A study did a comparison between all three situations (primary LNM (nodal metastasis at time of presentation) versus secondary LNM (nodal metastasis later after initial diagnosis) versus initial LNM in MUP) by Sondak et al. showed poorer survival of patients with primary LNM favoring patients with MUP over patients with MKP (metastatic melanoma with known primary) [12]. Another study done on 498 patients of malignant melanoma with nodal metastasis between 1996 and 2010 found that patients with secondary LNM and MUP patients had a considerably better prognosis compared to initially metastasized patients with known primary tumor both in bivariate and multivariable analyses [13].

Another reported case with the same criteria of our reported case was found after the literature review [14]. That case was managed with lymph node excision including complete ilioinguinal lymph node dissection. The management was composed of dacarbazine, vincristine, and cisplatin based chemotherapy along with radiotherapy to the regional nodal basin. The recovery was uneventful [14].

Our case was managed with surgical excision with subsequent appearance of a seroma at the place of the surgery that was managed with fine needle aspiration. Fluid obtained was sent for cytology analysis that revealed the same malignant melanoma cells. Patient was treated with radiation therapy session followed by cycles of ipilimumab, known to be an antibody to immune check point molecule CTLA4, which is used in metastatic malignant melanoma.

## 3. Conclusion

We present a case of malignant melanoma of unknown primary presenting in an unusual place which is the inguinal lymph node. Theories try to explain the pathway of development of such tumors and one of the theories mentions that it could be a spontaneous regression of the primary cutaneous lesion. Another theory is that it could be from transformation of aberrant melanocyte within the lymph node. Prognosis is postulated to be better in this case than in melanoma with a known primary.
